# Development of a Plant‐Based Chocolate Spread With Enhanced Vitamin D3 Bioavailability and Balanced Omega Fatty Acids

**DOI:** 10.1002/fsn3.70827

**Published:** 2025-09-07

**Authors:** Dina E. H. Azab, Ebtehal A. Altamim, Tarek N. Soliman, Sahar A. Nasser, Hamdy A. Zahran

**Affiliations:** ^1^ Food Technology Department, Food Industries and Nutrition Research Institute National Research Centre Cairo Egypt; ^2^ College of Sport Sciences and Physical Activity Princess Nourah Bint Abdulrahman University Riyadh Kingdom of Saudi Arabia; ^3^ Dairy Department, Food Industries and Nutrition Research Institute National Research Centre Cairo Egypt; ^4^ Food and Dairy Science and Technology Department, Faculty of Agriculture Damanhour University Damanhour Egypt; ^5^ Fats and Oils Department, Food Industries and Nutrition Research Institute National Research Centre Cairo Egypt

**Keywords:** bioavailability, chocolate spread, nano‐liposomal vitamin D_3_, omega‐3 fatty acids, plant‐based

## Abstract

This study developed a vegan chocolate spread using spray‐dried plant‐based milk powders (soy, lentil, and rice), fortified with nano‐liposomal vitamin D3 and an oleogel‐balanced omega fatty acid to enhance nutritional quality. The plant‐based milk powders exhibited high protein (up to 26.8% in soy), fiber, and micronutrients. They were successfully incorporated into chocolate spreads, yielding products with a favorable texture and sensory acceptance comparable to commercial spreads. Nano‐liposomal encapsulation of vitamin D3 achieved high encapsulation efficiency (94.2%), uniform nanoscale particle size (average 112 nm), and robust stability, ensuring effective vitamin delivery throughout storage. The oleogel, formulated from a blend of almond, coconut, avocado, red palm, and flaxseed oils, significantly improved the omega‐3 to omega‐6 fatty acid ratio (from 1:15 to 1:4.7) and provided enhanced oxidative stability, as confirmed by Rancimat testing. Sensory evaluation indicated that the fortified spreads maintained desirable flavor, mouthfeel, and overall acceptability. These results demonstrate that combining plant‐based milk powders, nano‐liposomal vitamin D3, and a balanced oleogel can produce a sustainable, nutritious, and appealing vegan chocolate spread. This approach offers a promising alternative for health‐conscious consumers seeking functional, plant‐based confectionery products.

## Graphical abstract

Developed a novel plant‐based chocolate spread using soy, lentil, and rice milk powders, offering a sustainable dairy alternative. Enhanced Vitamin D3 bioavailability through nano‐liposomal encapsulation, addressing a key micronutrient deficiency. Improved the fatty acid profile by incorporating an oleogel with balanced omega‐3 and omega‐6 fatty acids. The resulting spread caters to the growing demand for vegan, healthy, and sustainable confectionery options. Comprehensive analysis confirmed the chemical composition, oxidative stability, and characteristics of the fortified spread.

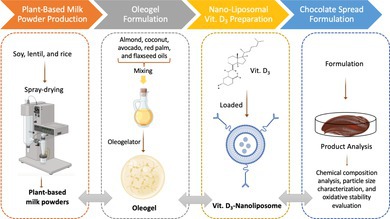

## 1 Introduction

The global shift toward plant‐based diets has sparked a surge in demand for sustainability. The growing demand for plant‐based alternatives to traditional dairy and confectionery products has driven the development of innovative food formulations that cater to the needs of health‐conscious consumers (McClements 2020). Among the most promising ingredients for these alternatives are rice, soy, and lentils, which offer unique nutritional profiles and functional properties (Nyambayo et al. 2024). Plant‐based milk alternatives, also known as “plant pints of milk,” have emerged as a popular choice, with the global market for these products expected to reach a value of $26 billion in the next 5 years (Mastromonaco et al. 2025). These plant‐based beverages, derived from the water extraction of legumes, nuts, or cereals, are often perceived as healthier, more sustainable, and more welfare‐friendly than their dairy counterparts (Plant‐based Milk Market to Reach $52.54 Billion by 2031, 2023). However, a potential drawback of plant‐based diets is that they may lack certain key micronutrients, such as vitamin B12, D, calcium, and omega‐3 fatty acids (Grasso et al. 2023). To address this, researchers have explored the development of next‐generation plant‐based chocolate spreads that are nutritionally fortified to provide a more complete nutritional profile (Taha et al. 2024).

Chocolate is a beloved indulgence for many, offering a unique flavor and texture that has made it a staple in the food industry (Tolve et al. 2022). However, the high concentrations of saturated fats and sugar found in traditional chocolate spreads have raised concerns among health‐conscious consumers, as these components contribute to the development of cardiovascular diseases and other metabolic disorders (Tolve et al. 2022). To address these concerns, researchers have explored the possibility of enhancing chocolate spread with beneficial nutrients, such as vitamin D3 and omega fatty acids, while maintaining the desirable sensory properties of the product.

Vitamin D3, also known as cholecalciferol, is a fat‐soluble vitamin that plays a crucial role in bone health, immune function, and cardiovascular function (Nehlig 2013). Unfortunately, many individuals do not obtain sufficient amounts of vitamin D3 from their diet or through sun exposure, making the fortification of food products a potentially effective strategy to address this deficiency. To improve the bioavailability and stability of vitamin D3 in chocolate spreads, researchers have investigated the use of nano‐liposomal encapsulation, a technique that involves the incorporation of the vitamin into tiny lipid‐based vesicles (Aggeletopoulou et al. 2024; Rahim et al. 2025).

In addition to vitamin D3, the inclusion of balanced omega fatty acids in chocolate spreads has also garnered significant interest. Omega‐3 and omega‐6 fatty acids are essential for various physiological processes, including brain function, inflammation regulation, and cardiovascular health. However, the typical Western diet often features an imbalance in the ratio of these fatty acids, with an excess of omega‐6 and a deficiency of omega‐3 (Mohammed et al. 2025). To address this imbalance and provide a more beneficial lipid profile, researchers have explored the use of oleogels, which are semi‐solid, lipid‐based structures that can be incorporated into chocolate spreads to modulate the overall fatty acid composition (Afoakwa 2010). By leveraging these innovative approaches, chocolate manufacturers can cater to the growing demand for healthier and more functional food options, ultimately improving the overall well‐being of consumers. While previous studies have explored plant‐based alternatives and nutrient fortification separately, a comprehensive approach combining plant‐based milk powders, nano‐liposomal vitamin D3, and oleogel‐balanced fatty acids in chocolate spreads has not been thoroughly investigated.

The incorporation of nano‐liposomal vitamin D3 and oleogel‐balanced omega fatty acids into chocolate spreads has the potential to enhance the nutritional profile of these products while maintaining their desirable sensory characteristics. Maintaining desirable sensory attributes, such as texture and flavor, is a critical consideration in the development of these novel plant‐based chocolate spreads. Therefore, this study hypothesizes that plant‐based milk powders derived from soy, lentils, and rice can be successfully incorporated into chocolate spreads, creating vegan alternatives with desirable sensory properties. Furthermore, the encapsulation of Vitamin D3 within nanoliposomes will effectively enhance its bioavailability and stability in the spread, while the incorporation of a carefully formulated oleogel will improve the overall fatty acid profile by increasing the omega‐3 to omega‐6 ratio. This research aims to develop and characterize these novel plant‐based chocolate spreads, evaluating their chemical composition, oxidative stability, and the properties of the incorporated nano‐liposomal Vitamin D3, thus providing a nutritious and sustainable alternative to traditional chocolate spreads.

## 2 Materials and Methods

### 2.1 Materials

Brown lentils Giza 9 ( *Lens culinaris* ), soybeans *Glycine max* (*L*.) *cv*. Giza 111, and rice ( *Oryza sativa* L.) Giza 171 were obtained from the Agricultural Research Center (ARC), Giza, Egypt. Vegetable oils were provided by Purity Co., Cairo, Egypt. Oleogelator (Oleofat) was donated by Oleo Misr Co., Sadat City, Egypt. Sugar, dark cocoa powder, and milk butter were purchased from a local market (Dreem, Cairo, Egypt). Vitamin D_3_ (*Cholecalciferol*) and all chemicals were of analytical grade and purchased from Merck, Germany.

### 2.2 Methods

#### 2.2.1 Preparation of Milk Powders

To produce milk powders, the raw materials—soybeans, brown lentils, and rice—were first cleaned, washed, and dried to remove impurities and excess starch (Goonathilaka et al. 2023). The detailed process for each material is as follows:

– Soybeans: Soaked in water at a 1:9 ratio, then ground to extract soymilk. The mixture was filtered through a muslin cloth (Taha et al. 2024).
– Brown Lentils: Soaked in water at a 3:7 ratio for 12 h, cooked at 100°C for 30 min, ground, and blended until smooth. The mixture was strained through a muslin cloth (Naeem et al. 2024).
– Rice: Soaked in water at a 1:4 ratio for 8 h, cooked at 100°C for 30 min, and the resulting slurry was filtered using a clean muslin cloth (Moradi et al. 2021).

The extracted plant‐based milks were then dried using a BUCHI Mini Spray Dryer B‐290 (Büchi Labortechnik AG, Flawil, Switzerland). The spray dryer's cylindrical chamber, approximately 500 mm in length and 150 mm in diameter, was operated with a liquid feed flow rate of 8 mL/min, a nozzle airflow of 440 L/h, and a drying airflow of 38 m^3^/h. The inlet air temperature was maintained at 160°C, with the outlet temperature not exceeding 90°C. Once dried, the powders were collected, passed through 20‐mesh sieves, and packed after cooling. This process is summarized in the following flow diagram:

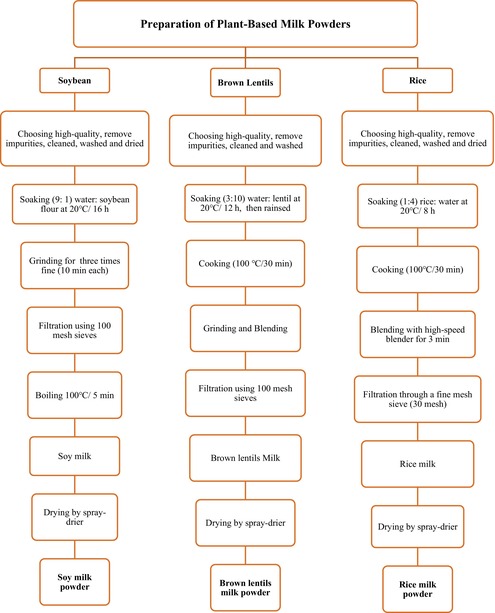

#### 2.2.2 Chemical Composition

According to the Association of Official Analytical Chemists' Official Methods of Analysis, the chemical makeup of three types of plant milk was evaluated for total solids, protein, and ash method No. 947.05 (AOAC 2016). The phenol‐sulfuric approach, described by DuBois et al. (1956), was used to quantify the amount of carbohydrates. This technique quantifies the total amount of hydrolyzable carbohydrates. The amount of crude fat was measured using the *Soxhlet* equipment (AnkomXT10 fat extractor, Ankom Technology, Macedon, NY, USA) and solvent extraction technique No. 30‐25, as described in (AACC 2000). The *Soxhlet* equipment was used to connect a thimble to a 2 g moisture‐free sample, which was then added to a 50 mL flask. By varying the rate of 3–4 drops of hexane per second, the sample was fat‐extracted for 2–3 h. Once the 6–7 siphons, the thimble was removed and put in an oven set at 100°C ± 5°C for 1 h before being weighed.

(1) $$\;\text{Crude}\;\mathrm{fat}\;\left(\%\right)=\frac{\text{loss in weight}\;\left(g\right)}{\text{weight of sample}\;\left(g\right)}\times 100$$

For crude fiber determination, the AOCS (2000) official method is used in samples with the use of a filter bag 128. Analyzed using the following formula and approved procedure Ba 6a‐05 (Ankom Technology Corp., Macedon, NY, USA):

(2) $$\%\text{Crude Fiber}=\frac{\text{Weight of oven dried residue}\;\left(g\right)-\text{Weight after ashing}\;\left(g\right)}{\text{Weight of sample free}\;\mathrm{fat}\;\left(g\right)}\times 100$$

#### 2.2.3 Nutritional Values of Three Plant‐Based Milk Powders

Proteins give 4 kcal/g; carbohydrates give 4 kcal/g; and fats give 9 kcal/g (Hall et al. 2015). The equation was used to compute energy:

(3) $$\text{Energy}\;\left(\text{Kcal}\right)=\text{carbohydrate}\;\left(g\right)\times 4+\text{protein}\;\left(g\right)\times 4+\mathrm{fat}\;\left(g\right)\times 9$$

#### 2.2.4 Preparation of Vitamin D3‐Loaded Liposomes

The thin‐layer hydration technique was employed to encapsulate vitamin D3 in liposomes, utilizing 50 μg of vitamin D3 and 0.1 g of soy lecithin mixed in 15 mL of ethanol. This mixture was subsequently placed in a vacuum rotary evaporator (Heidolph, Laborota 4002 control, Schwabach, Germany) within a round‐bottom flask at 123 mbar for 60 min at 30°C to eliminate all organic solvents and produce a thin, wet film at the flask's base. In a rotating apparatus at 30°C, a thin layer was hydrated with 10 mL of distilled water and 0.5 g of glass beads were added to the flask, followed by the use of a rotary evaporator (without vacuum). The dimensions of the original liposomes were diminished using a combination of homogenization (Sonic Vibra cell, Newton, CT, USA) at 20,000 rpm for 20 min, followed by ultrasonication for 15 min at 160 W power, 40 kHz frequency, and 50% pulse (Newtown, CT, USA). The elevation in temperature during ultrasonication was mitigated by placing the sample container within a larger beaker filled with ice.

##### 2.2.4.1 Characterization of Liposomal Nanoparticles

The polydispersity index (PDI), mean particle size diameter, and surface charge (zeta potential) of vitamin D3 nanoliposomes (Zetasizer Nano Z.S., Malvern Instruments, UK) were investigated using the dynamic light scattering (DLS) approach. Transmission electron microscopy (TEM) was used to examine and characterize the morphology of nanoliposomes. A JEOL JEM‐1400 plus TEM operating at an accelerating voltage of 100 kV and a magnification of 200,000× was used (Abu‐El Khair et al. 2023).

##### 2.2.4.2 Encapsulation Efficiency of D3‐Nano Liposomal

Encapsulation efficiency (EE) is defined as the percentage of VD3 entrapped in liposomal formulations compared to the original quantity of VD3 administered. The total concentration of VD3 in the liposomal formulations and the free VD3 in the supernatant (isolated via centrifugation) were quantified using an HPLC system, specifically an Agilent 1260 series HPLC (Santa Clara, California, USA), by the methodology established by Temova and Roškar (2016). The column used was an Agilent C18 (4.6 mm × 250 mm i.d., 5 μm). The mobile phase consisted of methanol and acetonitrile at a ratio of 65:35, with a flow rate of 1.2 mL/min. The injection volume for each sample solution was 10 μL. The DAD was calibrated at 264 nm. The column temperature was maintained at 40°C. Vitamin D3 was identified by comparing its retention duration (5.98 min) with that of the standard. The following equation computes the EE:

(4) $$\mathrm{EE}\left(\%\right)=\frac{\text{Encapsulated}\;\mathrm{Vit}.D3}{\text{Total}\;\mathrm{Vit}.D3}\times 100$$

#### 2.2.5 Oil Characterization

##### 2.2.5.1 Melting Point (°C)

After being melted, the oil sample was pure and impure‐free. The oil was added to a clean capillary tube with a 1 mm inner diameter and a length of 50–800 mm such that the oil was about 10 mm high in each tube (AOCS 1997). By melting it over a flame, one end of the tube was sealed. The glass beaker containing a suitable heating medium (a water bath) was filled with the capillary tubes. For precise temperature measurements, a thermometer was attached to the beaker. The temperature of the sample was raised gradually, around 0.5°C every minute. To provide a uniform dispersion of temperatures, the heating medium was gently shaken. The temperature recorded at which the sample starts to melt (a droplet appears on the tube's side) and when it melts completely.

##### 2.2.5.2 Acid Value (AV)

The standard ISO requirements for detecting the free fatty acid (FFA) content were adhered to (ISO 660 2020). More specifically, 10 g of each oil sample was combined with 50 mL of neutral ethanol. The resulting mixture was stirred and titrated with a 0.1 N sodium hydroxide (NaOH) solution using phenolphthalein as an indicator. The FFA concentration was expressed as the weight percentage of oleic acid (% w/w) in the oil samples.

(5) $$\mathrm{AV}\;\left(\mathrm{mg}/g\right)=\frac{\mathrm{mL}\;\text{of}\;\mathrm{KOH}\times N\times 56.11}{\text{Mass of sample}\;\left(g\right)}$$

##### 2.2.5.3 Peroxide Value (PV)

The peroxide value (PV) was measured using the methodology outlined in ISO 3960 (2017). For the analysis, 5 g of the oil sample was dissolved in a 3:2 volumetric mixture of acetic acid and chloroform solvent. This solution was then treated with a supersaturated potassium iodide (KI) solution. After letting the reaction run for 5 min in the dark, the mixture was titrated with a 0.01 N sodium thiosulfate (Na_2_S_2_O_3_) solution. We used starch as the indicator. The results were given as milliequivalents of peroxide per kilogram of oil, or mEq O2/kg oil.

(6) $$\mathrm{PV}\;\left(\mathrm{meq}./\mathrm{kg}\right)=\frac{\left(V1-V2\right)\times N\times 1000}{W}$$

where *V*1 is a volume of sodium thiosulfate solution consumed in the titration of the sample (mL), *V*2 is a volume of sodium thiosulfate consumed in the titration of the blank (mL), *N* is the normality of sodium thiosulfate, and *W* is the weight of the oil sample (g).

##### 2.2.5.4 Iodine Value (IV)

The iodine value (IV), which is a crucial metric for determining the level of unsaturation in vegetable oils, is the amount of iodine (in grams) that interacts with 100 g of oil. More unsaturated fatty acids are present in the oil as the IV rises. The IV was calculated based on the concentrations of certain unsaturated fatty acids using the method below (Gagour et al. 2024):

(7) $$\mathrm{IV}=\left(\%C_{16:1}\times 1.001\right)+\left(\%C_{18:1}\times 0.899\right)+\left(\%C_{18:2}\times 1.814\right)+\left(\%C_{18:3}\times 2.737\right)$$

##### 2.2.5.5 Composition of Fatty Acids in Oil Samples

By using Zahran and Tawfeuk's (2019) modified approach to convert oil into FAMEs, the fatty acid composition was determined. After vortexing 1.0 mL of n‐hexane and 15 mg of echium oil for 30 s, 1 mL of sodium methoxide (0.4 mol) was added, vortexed (30 s.), and allowed to settle (15 min). The upper phase containing FAME was recovered and analyzed using GC with a capillary column (Supelco SP‐2380 60 m × 0.25 mm × 0.20 μm; Sigma‐Aldrich, USA), detector (FID), and an HP 6890 plus gas chromatography (Hewlett Packard, USA); the FAMEs were separated. The injector and detector temperatures were set at 250°C. At 140°C for 5 min, the temperature in the column rose to 240°C at a rate of 4°C per minute, and it remained there for 10 min. Helium was the carrier gas, flowing at a rate of 1.2 mL min^−1^. By comparing their relative and absolute retention periods to those of genuine standards of FAMEs (SupelcoTM 37‐component FAME mix), FAMEs were discovered. The proportion of the entire peak area that was made up of fatty acids was reported.

#### 2.2.6 Oleogel Formulation

To ensure thorough mixing and full integration of the gelling agents, a blend of oils was made with 25% almond oil, 25% coconut oil, 20% avocado oil, 15% red palm oil, and 15% flaxseed oil. The oils were heated to 60°C to aid in blending, after which the oleogelator (Oleofat) was added at a 5% ratio (w/w) from the batch (200 g). The mixture was then homogenized for 120 s, cooled to form a stable oleogel, and prepared for easy incorporation into the chocolate mixture.

#### 2.2.7 Vegan Chocolate Spread (VOC) Preparation

Chocolate spread fortified with encapsulated D3, as described by Nur et al. (2021), features two modifications: fat sources and the type of milk powder. The ingredients include cocoa powder (19.5%), fat phase (27%), sugar (36%), whole milk powder (16.41%), vanilla (0.02%), and 0.0334% of vitamin D3 encapsulated in liposomes. Butter was used as the fat phase, while whole milk powder served as the basis for the control chocolate spread (CCS). To prepare the oleogel chocolate spread that acts as a control (COCS), oleogel at 27% was utilized as a fat alternative alongside the other three treatments. To develop vegan chocolate spread (VCS) treatments based on the modifications mentioned above, brown lentils, rice, or soy milk powder were incorporated at 16.41% each, acting as milk alternatives for three specific formulations: soy vegan chocolate spread (SVCS), lentil vegan chocolate spread (LVCS), and rice vegan chocolate spread (RVCS).

#### 2.2.8 Oxidative Stability (Rancimat Test)

The oxidative stability (OS) of the oil samples was evaluated using the Rancimat test by ISO standard procedures (ISO 6886 2016). This method measures OS using the oxidative induction time (in hours). Each oil sample was weighed at three grams and placed in the reaction vessel of the Rancimat instrument (890 Metrohm, Herisau, Switzerland). The tank was heated to 110°C, and the airflow rate was adjusted to 20 L/h. The volatile compounds generated during the oxidation process were collected in a flask filled with 60 mL of deionized water. The conductivity of this solution, which increases as the amount of volatile oxidation products increases, was continuously observed and recorded using the Rancimat application.

#### 2.2.9 Color of Vegan Chocolate Spread

Color parameters were carried out for the vegan chocolate samples by Azab and Mahmoud (2024). CIE L*, a*, and b* parameters were evaluated using a Hunter colorimeter (Hunter Lab Scan XE, USA), where C*: Chroma and WI: whitening index were calculated from CIE parameters using the equations as follows:

(8) $$C^{*}=\sqrt{a^{*2}+b^{*2}}$$

(9) $$\mathrm{WI}=\sqrt{a+b+{\left(100-L\right)}^{2}}$$

#### 2.2.10 Rheology of Chocolate Spread

##### 2.2.10.1 Viscosity

The viscosity of the chocolate spread samples was measured using a Brookfield viscometer (Middleboro, MA, USA) equipped with a concentric cylinder probe. Shear rates ranging from 1 to 100 s^−1^ were applied at a temperature of 23°C. The flow properties of the vegan chocolate spread samples were analyzed using the power‐law equation to determine key parameters, including the consistency coefficient (*K*) and the flow behavior index (*n*).

The power‐law equation used was

(10) $$\tau =K\cdot \gamma^{n}$$

where

- τ represents shear stress (Pa),
- γ**˙** is the shear rate (s^−1^),
- *n* is the flow behavior index.

This analysis was conducted to assess the non‐Newtonian flow behavior of the samples, following the methodology described by Tolve et al. (2021).

##### 2.2.10.2 Texture Profile Analysis

All texture parameters of the chocolate spread samples were determined by a conical probe (texture analyzer, TAHD Plus, Stable Micro System, Godalming, UK) with a compression force of 55 mm and a speed of 1 mm/s, according to Azab and Mahmoud (2024).

#### 2.2.11 Sensory Acceptability

Sensory evaluation was according to Furlán et al. (2017) with some modifications. Chocolate spread samples were coded with a three‐digit code and randomly serialized. The samples were tested at room temperature by trained panelists in the Department of Food Industries and Nutrition Institute at the National Research Centre in Egypt. The panelists judged the samples on a ten‐point hedonic scale (10 = extremely like, 5 = neither like nor dislike, 1 = extremely dislike). Color, taste, texture, appearance, aroma, smoothness, and overall acceptability were evaluated.

### 2.3 Statistical Analysis

Statistical analysis was conducted using the GLM procedure as described by Rodríguez‐Estévez et al. (2011). Each value is presented as the mean ± standard deviation (SD) based on six replicates (*n* = 6). To compare means, analysis of variance (ANOVA) and Duncan's multiple comparison tests were performed using SPSS software (version 22.0, SPSS Inc., Chicago, IL, USA). Statistical significance was determined at a probability level of *p* ≤ 0.05.

## 3 Results and Discussion

### 3.1 Chemical Composition and Nutritional Value of Powdered Milk of Rice, Soy, and Brown Lentil

The (Table 1) shows that brown lentil milk powder is highest in ash (5.85%) and protein (30.88%), while soybean milk powder has the highest fat (23.66%) and energy (490.78 Kcal), and rice milk powder stands out for its very high carbohydrate content (78.58%) but lowest protein (9.87%). Moisture content is similar across all samples, with rice milk powder slightly lowest (3.61%).

The nutrition profiles of these plant‐based milk powders reveal that soymilk is especially rich in protein and fat, positioning it as a preferable alternative to traditional dairy. The protein quality in soymilk is comparable to that of animal proteins, benefiting health‐minded consumers (Asaduzzaman et al. 2023). Furthermore, soymilk has been linked to various health advantages, such as potential reductions in the risk of cardiovascular disease and enhancements in bone health due to its isoflavone content (Craig and Fresán 2021). The high‐fat content in soymilk, derived mainly from polyunsaturated fatty acids, boosts its energy density and nutritional value, which is vital for those exploring plant‐based diets (Taşoyan and Yolaçaner 2023). While brown lentils have less fat than soymilk, they offer significant amounts of protein and carbohydrates, making them an essential nutritional source, especially for vegetarian and vegan diets (Singh et al. 2020). Although rice milk is high in carbohydrates and provides energy, it may lack the protein or fat levels necessary for a balanced diet (Vanga and Raghavan 2017). The nutritional variances among these plant‐based milk powders stress the importance of choosing the right type according to dietary requirements and health objectives. The chemical composition and nutritional benefits of powdered milk from brown lentils, soybeans, and rice highlight the variety of plant‐based alternatives available. Each type offers distinct advantages, with soymilk excelling in protein and fat content.

### 3.2 Vitamin D3‐Liposome Encapsulated to Be Applied in Vegan Chocolate Spread

**FIGURE 1:**
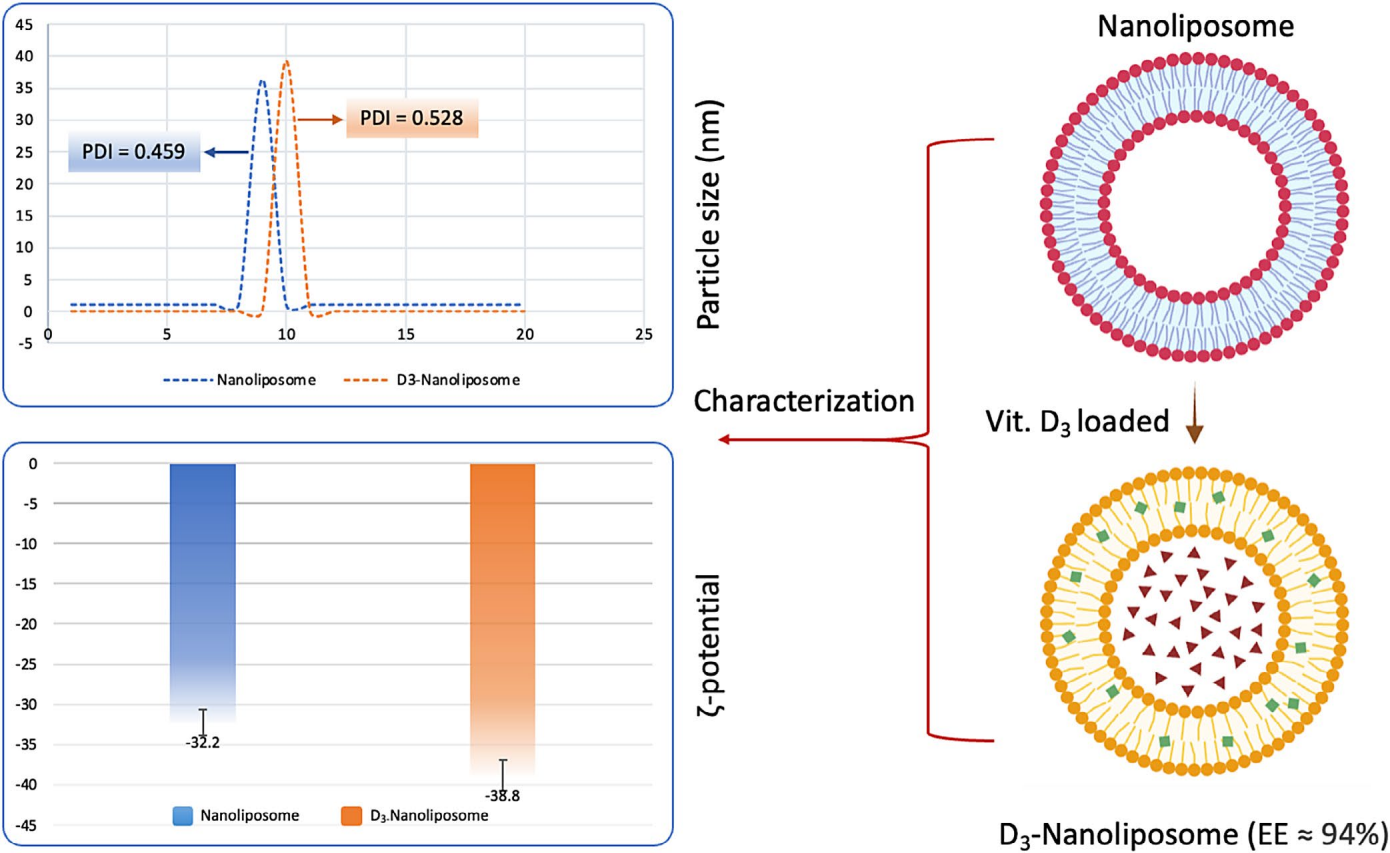
Average particle size, Polydispersity Index (PDI), surface charge (ζ‐potential) of nanoliposomes, and D3 loaded on nanoliposomes.

Encapsulating Vitamin D3 in liposomes for use in a vegan chocolate spread is an intriguing application of nanotechnology in food science. Figure 1 presents the average particle size, Polydispersity Index (PDI), and zeta potential of standard and D3‐loaded nanoliposomes. The D3 nanoliposomes show a slight increase in particle size compared to the plain nanoliposomes, suggesting successful encapsulation of Vitamin D3 within the liposomal structure. Ghanbarzadeh et al. (2016) found the same trend when loading phycocyanin in liposomes; the particle size increased with increases in the addition of phycocyanin. Particle sizes between 20 and 200 nm are ideal for enhancing solubility and absorption in the gastrointestinal tract (Mohammadi et al. 2014).

**TABLE 1:** Chemical composition and nutritional value of powdered milk of rice, soy, and brown lentils.

Sample	Moisture (%)	Ash (%)	Protein (%)	Fat (%)	Carbohydrate* (%)	Energy (Kcal)
*L*	4.82^a^ ± 0.10	5.85^a^ ± 0.14	30.88^b^ ± 0.17	4.44^b^ ± 0.14	53.99^b^ ± 0.62	379.44^b^ ± 4.42
*S*	3.31^c^ ± 0.12	3.57^c^ ± 0.08	33.54^a^ ± 0.27	23.66^a^ ± 0.34	35.92^c^ ± 0.54	490.78^a^ ± 6.30
*R*	3.61^b^ ± 0.05	4.17^b^ ± 0.13^a^	9.87 ^c^ ± 0.68	3.77^c^ ± 0.51	78.58^a^ ± 0.36	387.73^b^ ± 8.75

*Note:* The results were expressed as mean ± SD (*n*‐values = 3); different letters in the same column indicate significant differences between samples. Total carbohydrate was calculated by difference.

Abbreviations: L, brown lentil milk powder; R, rice milk powder; S, soybean milk powder.

The PDI value for plain nanoliposomes is 0.459, indicating a relatively narrow size distribution. D3‐nanoliposomes increase to 0.528, reflecting heterogeneity possibly due to aggregation or inconsistencies during vitamin integration (Mohammadi et al. 2014). Uniform size distribution is crucial for product quality and stability. The increase in PDI for the D3‐nanoliposome reflects a change in size distribution upon loading the vitamin, possibly due to aggregation or inconsistencies during D3 integration. Since uniform size distribution is critical for product quality and stability, this increase may necessitate further optimization in the formulation process. Zeta potential measures the stability of colloidal dispersions. The D3‐nanoliposome has a more negative zeta potential than the plain nanoliposome, indicating enhanced electrostatic repulsion among particles, which leads to improved stability. The EE of 93.76% shows that a significant proportion of Vitamin D3 is successfully incorporated into the nanoliposomes. This benefits food products, ensuring that a substantial amount of the vitamin remains available for absorption without significant degradation during processing or storage. Encapsulating Vitamin D3 in liposomes for use in a vegan chocolate spread offers several advantages, including small particle size and effective encapsulation that can enhance the bioavailability of Vitamin D3, making it more accessible for absorption in the body. Furthermore, improved stability from the negative zeta potential means the product is less likely to separate or degrade over time, preserving a consistent flavor and texture. A vegan chocolate spread enriched with Vitamin D3 can appeal to health‐conscious consumers searching for functional foods.

### 3.3 Morphological Properties of D_3_ Liposomes by TEM

**FIGURE 2:**
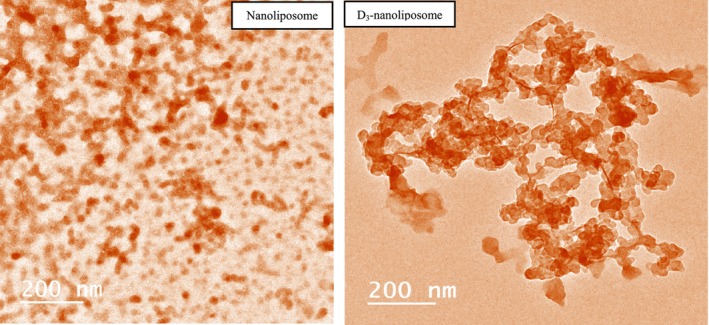
Micrograph of liposome nanoparticles and D_3_ loaded into liposomes.

Studying D_3_‐loaded liposomes, particularly through the lens of morphology and microstructure, can significantly enhance our understanding of their physical characteristics and stability. TEM is a critical methodology for visualizing liposomal constructs. Morphologically, D3‐loaded liposomes can exhibit various structural forms, commonly appearing as spherical vesicles. TEM images frequently reveal a well‐organized lipid bilayer, confirming the encapsulation of D_3_ within lipid assemblies (Figure 2). For example, the negative staining technique is often employed to increase contrast in TEM observations, allowing for more precise visualization of the liposome structure, which is essential for effective drug delivery systems (Pisani et al. 2022).

The consistency of size and shape between dynamic light scattering (DLS) and TEM contributes to the characterizations' reliability. Typically, DLS results suggest a hydrodynamic diameter, while TEM offers insights into the actual morphology of the lipid bilayers (Pisani et al. 2022). Noteworthy is that some studies report an average hydrodynamic diameter of liposomes around 39.30 nm as measured by DLS (Pisani et al. 2022). The particle sizes measured via TEM are slightly smaller than those obtained through DLS. This difference may originate from the sample preparation processes involved in TEM, which can induce slight vesicle shrinkage (Yanar et al. 2020).

### 3.4 The Significance of the Blend's Replaced Plant Oils

The carefully chosen mix of plant‐based oils that make up the suggested oil blend for the spreadable, healthy chocolate replaces animal fats with distinct nutritional and functional advantages. The suggested oil ratios for the intended formula are shown in the accompanying diagram:

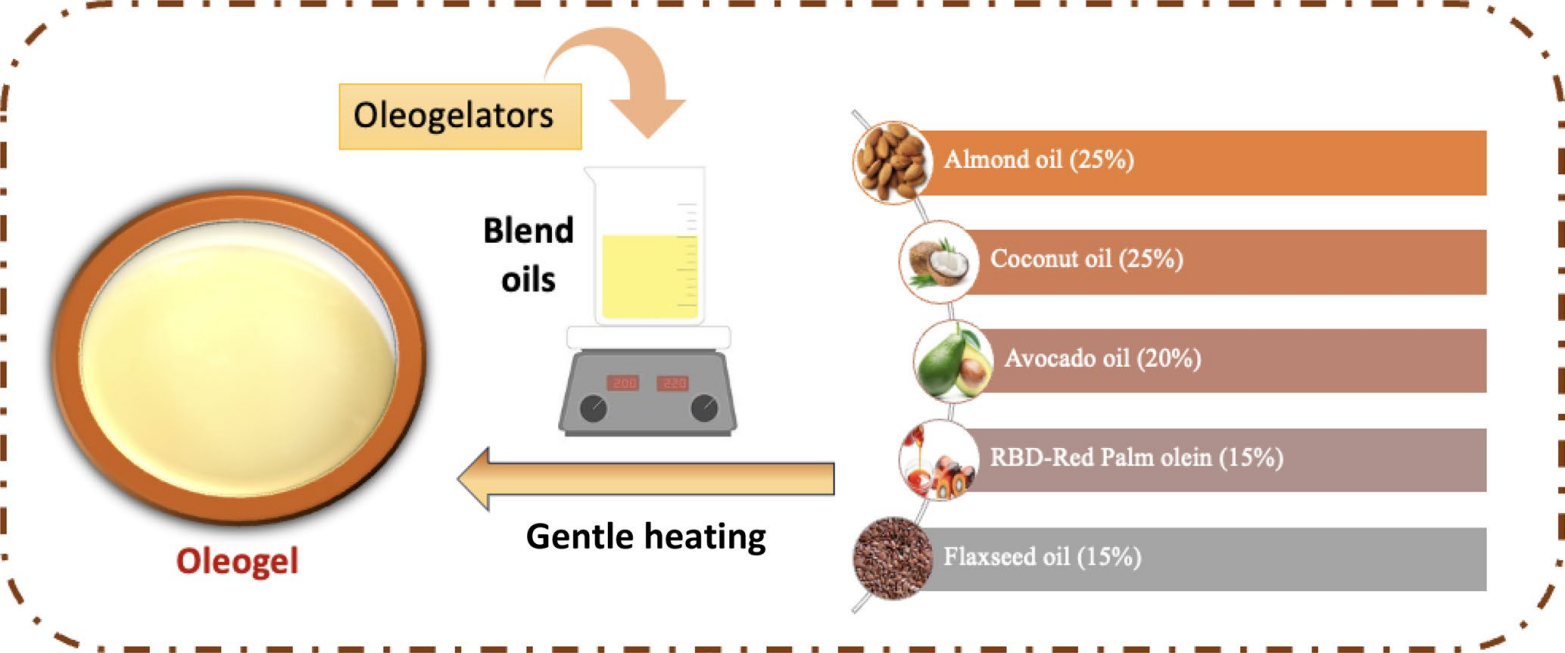

**Flow diagram of suggested oil ratios for the intended formula**.

According to El Idrissi et al. (2024), almond oil (25%) has a high content of monounsaturated fats and vitamin E, which may improve heart health and balance out the rich taste of the chocolate. Avocado oil (20%) mimics the feeling of butter further by adding smoothness and being high in antioxidants (Doan et al. 2016). Medium‐chain triglycerides (MCTs) from coconut oil (25%) provide instant energy, harden the chocolate, and provide a delicious taste (Sim et al. 2016). Because of its balanced fatty acid composition, palm oil (15%) improves stability and texture; nevertheless, to meet environmental problems, it must be sourced responsibly (Hosseinzadeh‐Bandbafha et al. 2022). Finally, 15% flaxseed oil has a subtle taste that balances the mix and is a great source of omega‐3 fatty acids that support heart health and reduce inflammation (Saidaiah et al. 2024). The carefully chosen combination of oils not only successfully substitutes animal fat but also improves the spreadable chocolate's nutritional value. Every oil has special qualities that enhance taste and texture, provide vital fatty acids, and promote heart health. This recipe guarantees a tasty result that satisfies vegan requirements while also keeping up with the current trend toward plant‐based diets.

### 3.5 Quality Parameters of the Individual Oils and the Oleogel

Important information about the functional characteristics and overall performance of the component oils and the oleogel made from them for use in spreadable chocolate may be gleaned from the quality metrics of these oils. Important parameters that are necessary for evaluating the stability and quality of oil are included in Table 2, including the acid value, peroxide value, iodine number, and melting point.

**TABLE 2:** Oils' quality characteristics which used for spreadable chocolate preparation.

Parameter	Almond oil	Avocado oil	Coconut oil	RBD‐Red palm olein	Flaxseed oil	Mix oils (oleogel)	Butterfat
Acid value (mg/g)	0.53 ± 0.02	0.64 ± 0.01	0.24 ± 0.01	0.16 ± 0.01	0.93 ± 0.03	0.58 ± 0.01	1.64 ± 0.22
Peroxide value (meq. O2/kg)	0.78 ± 0.11	1.02 ± 0.17	0.47 ± 0.02	0.32 ± 0.02	2.18 ± 0.13	0.96 ± 0.05	2.65 ± 0.16
Iodine number	133.77 ± 2.64	101.00 ± 1.17	24.32 ± 1.22	62.47 ± 1.37	184.39 ± 3.15	92.70 ± 1.14	39.27 ± 0.11
Melting point (°C)	−11.5 ± 1.0	−5.8 ± 0.5	33.7 ± 1.0	28.2 ± 0.7	−21.8 ± 0.05	26.2 ± 0.5	32.72 ± 0.74

*Note:* The results were expressed as mean ± SD (*n*‐values = 3).

Abbreviation: ND, not detectable.

The amount of free fatty acids in the oils is indicated by the acid value, which might affect shelf life and taste. Out of all the oils in this mix, flaxseed oil has the highest acid value (0.93 ± 0.03), indicating a higher risk of rancidity. With the lowest acid value (0.24 ± 0.01), coconut oil is the most stable and has a longer shelf life. In food applications, lower acid levels are often chosen to preserve quality over time (Marra et al. 2023). Lower readings imply higher oil quality. The peroxide value reflects the level of lipid oxidation (Rehim et al. 2023). Once again, flaxseed oil has the greatest peroxide value (2.18 ± 0.13), suggesting a greater vulnerability to oxidation. The oleogel's total peroxide value of 0.96 ± 0.05 indicates a balance between the oils, indicating enhanced oxidative stability as a result of the blend of oils with various oxidative characteristics. It has been shown that oleogels improve food goods' oxidative stability, extending their shelf life (Hwang 2020). Higher iodine values imply more unsaturation, which might affect texture and health benefits. The iodine value represents the degree of unsaturation in the oils (Zahran et al. 2021). With an iodine value of 184.39 ± 3.15, flaxseed oil contains the greatest concentration of polyunsaturated fatty acids, especially omega‐3's, which are good for heart health. Conversely, the low iodine value (24.32 ± 1.22) of coconut oil is indicative of its increased saturated fat content, which gives the oleogel a harder texture.

To control the oleogel's texture and spreadability in chocolate formulations, it is essential to ascertain its melting point. According to Boateng et al. (2016), flaxseed oil has a low melting point of −21.8°C ± 0.05°C, which contributes to a softer texture, whereas coconut oil has a comparatively high melting point of 33.7°C ± 1.0°C, helping to preserve firmness at room temperature. This mixture enables the finished product to have the desired uniformity.

Butterfat, with an acid value of 1.64 ± 0.22 mg/g and a peroxide value of 2.65 ± 0.16 meq. O_2_/kg, indicates a higher level of free fatty acids and oxidative degradation compared to most plant‐based oils. Its iodine number of 39.27 ± 0.11 suggests a moderate level of unsaturation, which is lower than that of flaxseed oil but higher than that of coconut oil. The melting point of butterfat is 32.72°C ± 0.74°C, which is close to that of coconut oil and oleogel, contributing to a firm texture at room temperature. In contrast, the oleogel, with an acid value of 0.58 ± 0.01 mg/g and a peroxide value of 0.96 ± 0.05 meq. O_2_/kg, an iodine number of 92.70 ± 1.14, and a melting point of 26.2°C ± 0.5°C, presents a balanced profile that combines stability with desirable sensory attributes. This comparison highlights the potential benefits of using plant‐based oleogels in vegan chocolate formulations, offering improved oxidative stability and nutritional profiles compared to traditional butterfat.

The chosen combination of oils not only successfully substitutes animal fat but also improves the spreadable chocolate's nutritional value and maximizes its sensory qualities by striking a balance between texture, taste, and stability. Regarding vegan chocolate applications, the quality criteria of each oil are crucial in guaranteeing that the finished product fulfills functional and sensory requirements.

### 3.6 Individual Oils' Fatty Acid Profiles and the Oleogel Made From Them for Spreadable Chocolate

**FIGURE 3:**
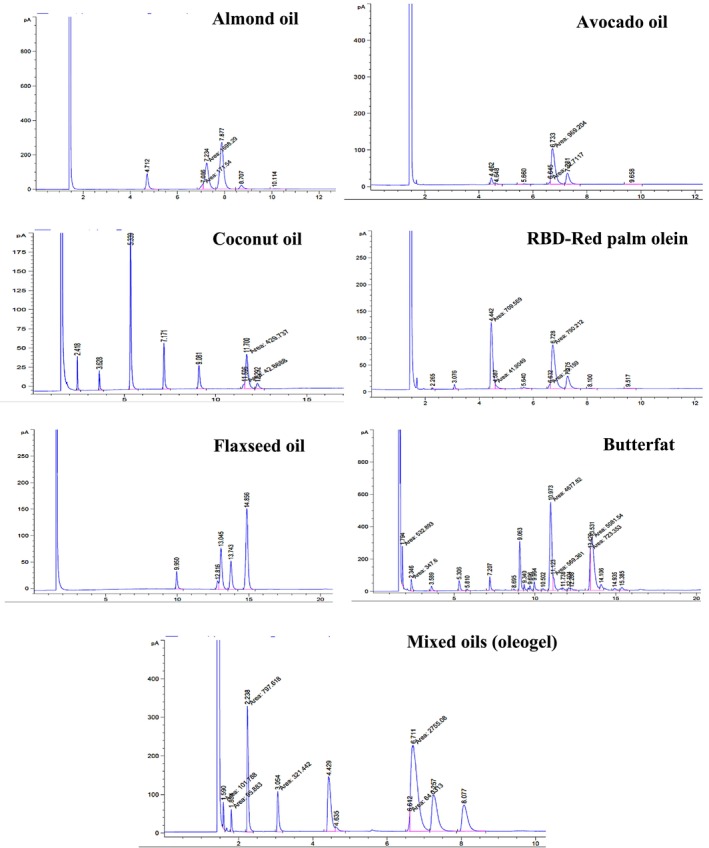
Chromatograms of individual oils and the oleogel fatty acids.

Important information about the nutritional and functional characteristics of each oil is provided by analyzing the fatty acid content of the individual oils and the oleogel made from these oils for use in spreadable chocolate. Table 3 and Figure 3 show several types of fatty acids that are necessary to comprehend the final product's textural characteristics and potential health effects. These include omega‐3, omega‐6, and unsaturated fatty acids (UFA), as well as saturated fatty acids (SFA) (Nguyen et al. 2019). The level of saturated fatty acids in the oleogel is modest at 33.28%. The main sources of these fatty acids are coconut oil (76.21%) and palm oil (42.37%). Elevated saturated fat content may help give the oleogel a more stable and hard texture, which is good for spreadable applications. However, because consuming too much saturated fat raises the risk of cardiovascular disease, the formulation must follow a balanced approach to guarantee health advantages while preserving desired sensory qualities (Nguyen et al. 2019).

**TABLE 3:** Fatty acid profiles for the oils used in spreadable chocolate preparation.

Fatty acids	RT	Almond oil	Avocado oil	Coconut oil	RBD‐Red palm olein	Flaxseed oil	Mixed oils (oleogel)	Butterfat
		Area (%)						
Butyric acid (C4:0)	1.79	ND	ND	ND	ND	ND	ND	3.26
Caproic acid (C6:0)	2.35	ND	ND	ND	ND	ND	ND	2.13
Caprylic acid (C8:0)	3.59	ND	ND	4.41	ND	ND	1.5	1.05
Capric acid (C10:0)	5.31	ND	ND	3.71	ND	ND	1.42	2.21
Umdecanoic acid (C11:0)	5.81	ND	ND	ND	ND	ND	ND	0.21
Lauric acid (C12:0)	7.21	ND	ND	43.99	0.48	ND	11.79	2.61
Tridecanoic acid (C13:0)	8.70	ND	ND	ND	ND	ND	ND	0.23
Myristic acid (C14:0)	9.06	ND	ND	12.8	1.38	ND	4.75	10.44
Myristoleic acid (14.1)	9.34	ND	ND	ND	ND	ND	ND	1.83
Pentadecanoic acid (C15:0)	9.70	ND	ND	ND	ND	ND	ND	1.71
Cis‐10‐Pentadecenoic (C15:1)	9.96	ND	ND	ND	ND	ND	ND	0.45
Palmitic acid (C16:0)	10.97	9.67	6.45	9.25	38.86	6.75	12.87	28.61
Palmitoleic acid (C16:1n7)	11.12	ND	ND	ND	ND	ND	ND	3.48
Palmitoleic acid (C16:1), n9	11.73	ND	0.95	ND	2.29	ND	0.92	0.72
Heptadecanoic acid (C17:0)	12.10	ND	ND	ND	ND	ND	ND	0.7
Cis‐10‐Heptadecanoic acid (C17:1)	12.27	ND	ND	ND	ND	ND	ND	0.7
Stearic acid (C18:0)	13.43	3.61	1.66	2.05	1.65	4.04	0.95	4.42
Oleic acid (C18:1), n9	13.53	29.7	70.91	20.59	43.96	24.43	40.66	31.08
Linoleic acid (C18:2), n6	14.11	53.08	19.36	3.2	10.89	16.12	14.72	2.19
α‐ Linolenic acid (C18:3), n3	14.94	3.94	0.67	ND	0.49	48.66	10.42	0.75
γ‐ Linolenic acid (C18:3n6)	15.39	ND	ND	ND	ND	ND	ND	1.22
SFA	—	13.28	8.11	76.21	42.37	10.79	33.28	60.36
UFA	—	86.72	91.89	23.79	57.63	89.21	66.72	39.64
Omega‐9 FA	—	29.7	71.86	20.59	46.25	24.43	41.58	34.78
Omega‐6 FA	—	53.08	19.36	3.2	10.89	16.12	14.72	3.41
Omega‐3 FA	—	3.94	0.67	ND	0.49	48.66	10.42	0.75
SFA/UFA	—	0.15	0.09	3.20	0.74	0.12	0.50	1.52

Abbreviations: FA, Fatty acid; ND, not detectable; RT, retention time; SFA, saturated fatty acids; UFA, unsaturated fatty acids.

High amounts of unsaturated fatty acids (66.72%) are found in the oleogel, primarily in almond oil (86.72%) and avocado oil (91.89%). Monounsaturated fats, especially oleic acid, which has been linked to heart health advantages, are abundant in these oils. In addition to improving spreadability and providing vital fatty acids that promote general health, the high UFA concentration gives the texture greater fluidity (Silva et al. 2022). Omega‐6 fatty acids make up 14.72% of the oleogel, with the majority coming from avocado oil (19.36%) and almond oil (53.08%). Although omega‐6 fatty acids are necessary for many body processes, an excess of omega‐3 fatty acids may cause inflammation. The World Health Organization (WHO) recommends maintaining an omega‐6 to omega‐3 ratio of around 4:1; hence, it is important to keep an eye on the formulation's omega‐6 concentration.

The addition of butter fat as a reference or control sample in the fatty acid profile (Table 3) provides a comprehensive comparison with the plant‐based oils and oleogel used in spreadable chocolate formulations. Butterfat exhibits a distinct fatty acid composition, with significant amounts of saturated fatty acids (SFA), such as palmitic acid (28.61%) and stearic acid (4.42%), contributing to its firm texture and stability. In contrast, the oleogel has a more balanced SFA/UFA ratio of 0.50, with a higher content of unsaturated fatty acids (UFA), particularly oleic acid (40.66%) and linoleic acid (14.72%). The oleogel also contains a notable amount of omega‐3 fatty acids (10.42%), primarily from flaxseed oil, which enhances its nutritional profile. Butter fat, on the other hand, has lower levels of omega‐3 fatty acids (0.75%) and a higher SFA/UFA ratio of 1.52, indicating a different nutritional and textural profile compared to the oleogel. This comparison highlights the potential benefits of using plant‐based oleogels in vegan chocolate formulations, offering improved nutritional profiles and sensory attributes compared to traditional butter fat.

The oleogel's omega‐3 content is mostly derived from flaxseed oil (10.42%), which also provides alpha‐linolenic acid (ALA), an essential nutrient for cardiovascular health and anti‐inflammatory properties. The oleogel's ratio of omega‐3 to omega‐6 fatty acids may lessen the risk of inflammatory reactions brought on by a high omega‐6 diet (Hamed et al. 2020). The oleogel's SFA/UFA ratio of 0.50 indicates a well‐balanced composition that incorporates the essential saturated fats for texture and stability while giving preference to unsaturated fats. This ratio is in line with the most recent dietary guidelines, which support consuming more unsaturated fats without totally cutting out saturated fats to support heart health (Huang et al. 2019; Iman et al. 2025). The nutritional profile and sensory qualities of the spreadable chocolate are largely determined by the fatty acid content of the various oils and the oleogel that is produced. Through meticulous selection and mixing of these oils, a product may be created that balances the fatty acid ratios to promote health goals while also satisfying customer preferences for flavor and texture. By using the advantages of both saturated and unsaturated fats, the composition guarantees a wholesome substitute for conventional spreads made with animal fat.

### 3.7 Physicochemical Properties of Vegan Chocolate Spread

Table 4 shows that incorporating oleogel into the formulation of chocolate spreads, particularly in the chocolate spread with oleogel prepared as a butter alternative (COCS), significantly impacts the product's color characteristics. In a comparative study of color parameters, the COCS sample exhibits a lightness (L*) value that suggests a reduction in lightness compared to traditional formulations, which can largely be attributed to the oleogel's unique ability to alter the structural and compositional properties of the chocolate spread, influencing how light interacts with the components in the matrix (Bascuas et al. 2021; Pușcaș et al. 2022). Research indicates that the structural changes initiated by oleogel, primarily derived from plant oils, modify texture and affect how cocoa solids disperse within the fat matrix, thereby impacting overall color (Ishak et al. 2024).

**TABLE 4:** Color parameter of the novel vegan chocolate spreads and the control samples.

Samples	L*	a*	b*	C*	WI
CCS	12.22^a^ ± 0.265	−0.089^b^ ± 0.003	0.45^a^ ± 0.026	0.459^b^ ± 0.026	9.37^c^ ± 0.12
COCS	12.00^a^ ± 0.195	−0.087^b^ ± 0.002	0.47^a^ ± 0.021	0.495^b^ ± 0.024	9.38^c^ ± 0.13
RVCS	1.613^d^ ± 0.05	−0.51^c^ ± 0.015	0.089^c^ ± 0.001	0.514^b^ ± 0.015	9.92^a^ ± 0.05
SVCS	9.37^b^ ± 0.32	−0.18^b^ ± 0.022	0.337^b^ ± 0.015	0.38^c^ ± 0.005	9.52^b^ ± 0.13
LVCS	6.46^c^ ± 0.20	0.38^a^ ± 0.015	0.52^a^ ± 0.010	0.642^a^ ± 0.007	9.67^b^ ± 0.10

*Note:* Mean ± SD was used to display the data (*n* = 3). A substantial variation (*p* < 0.05) was indicated by distinct letters for each column.

Abbreviations: CCS, control chocolate spread; COCS, chocolate spread with oleo‐gel prepared as a butter alternative; RCLS, vegan chocolate spread made with brown lentil milk powder and oleo‐gel as alternatives to whole milk powder and butter; RVCS, vegan chocolate spread made with rice milk powder and oleo‐gel as alternatives to whole milk powder and butter; SVCS, vegan chocolate spread made with soy milk powder and oleo‐gel as alternatives to whole milk powder and butter.

The oleogel's interfacial properties are critical in controlling the distribution of cocoa particles, potentially leading to a more homogeneous color, which informs consumer perception (Peixoto et al. 2025). Moreover, the oleogels emphasize modifying the rheological properties of chocolate spreads, subsequently influencing sensory attributes, including color perception (Pușcaș et al. 2022). The noted differences in lightness between COCS and other spreads indicate variations in appearance that could impact consumer acceptance, as lighter hues are often associated with higher quality in chocolate products (Bascuas et al. 2021).

Additionally, the type of milk powder incorporated into vegan chocolate spreads influences the product's color characteristics. For instance, a chocolate spread made with rice milk powder and oleogel (RVCS) showcases a notably lower *L* value than other formulations, indicating a darker color. This shift is influenced by the inherent properties of rice milk powder, which include specific pigments and a lower fat content than traditional dairy powders (Zhang et al. 2024). Examining the *a* and *b* values, RVCS presents a value indicative of a greenish hue, contrasting with the relatively neutral color profiles of other spreads (Zunjarrao and Fedorovskaya 2024).

Comparative analysis of soy milk powder‐based chocolate spread (SVCS) and brown lentil milk powder‐based spread (LVCS) further illustrates the impact of milk powder on color dynamics. The SVCS exhibited stable coloration, while the LVCS indicated a shift toward a redder hue, highlighting how different plant‐based milk powders can affect both color and sensory profiles (Doan et al. 2016; Soares et al. 2025). This underscores that formulation choices, particularly those related to milk powder, can significantly influence aesthetic and sensory attributes, thereby affecting marketability (Savchina et al. 2025).

The whiteness index (WI) represents another parameter critical to the visual appeal of chocolate spreads. For example, WI values suggest that these vegan formulations may resonate particularly well with health‐conscious consumers seeking appealing plant‐based alternatives (Patel and Dewettinck 2016). Incorporating oleogel and selecting milk powder significantly affect the color parameters of vegan chocolate spreads. Research demonstrates that oleogel contributes to a darker appearance by modifying lightness and color distribution, while the specific type of milk powder alters these effects based on its unique properties (Quispe‐Sánchez et al. 2022; Ishak et al. 2024). Understanding these interactions is essential for successfully formulating vegan chocolate spreads that align with consumer expectations for visual and sensory attributes.

### 3.8 Rheological Properties of Chocolate Spread

**FIGURE 4:**
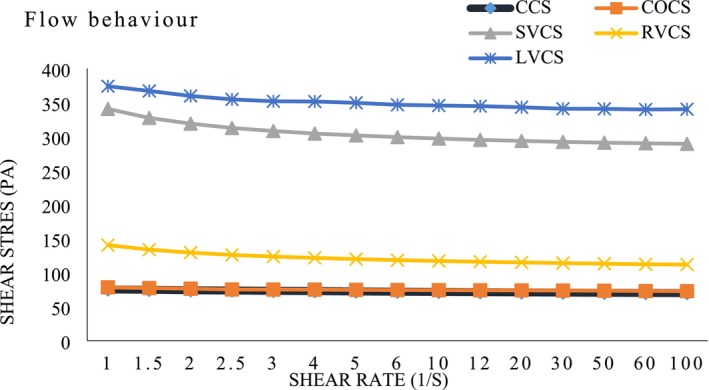
Flow behavior of the novel vegan chocolate spreads and the control samples. CCS, Control chocolate spread; COCS, Chocolate spread with oleo‐gel prepared as a butter alternative; LVCS, Vegan chocolate spread made with brown lentil milk powder and oleo‐gel as alternatives to whole milk powder and butter; RVCS, Vegan chocolate spread made with rice milk powder and oleo‐gel as alternatives to whole milk powder and butter; SVCS, Vegan chocolate spread made with soy milk powder and oleo‐gel as alternatives to whole milk powder and butter.

The viscosity of chocolate spreads is a crucial attribute that significantly influences their texture, mouthfeel, and consumer acceptance. Figure 4 illustrates the rheological properties of different chocolate spreads, including the control chocolate spread (CCS), the chocolate spread with oleogel (COCS), and various vegan formulations. These vegan options consist of the SVCS, the RVCS, and the LVCS. These marked differences are based on their compositions and ingredient types (Bascuas et al. 2021; Mohammadi et al. 2021). The flow curves generated from the shear stress and shear rate data indicate that the formulations exhibit nonlinear behavior characteristic of non‐Newtonian fluids, a common trait found in chocolate products (Kouřilová et al. 2022). When assessing the flow behavior, the LVCS samples achieved the highest shear stress at 373.81 Pa, compared with the CCS and COCS, which had 75.24 and 86.36 Pa, respectively. The RVCS and SVCS samples yielded 340.48 and 3.40 Pa shear stresses. This divergence suggests that including oleogel does not significantly elevate shear stress compared to conventional formulations, indicating that oleogel may facilitate a smoother texture without impairing spreadability (Pușcaș et al. 2022). Higher shear stress values observed in the LVCS and SVCS formulations can be linked to increased fiber and protein content of plant‐based milk powders. These components can enhance the thickening properties of the spreads, contributing to higher viscosity and increased structural complexity within the formulations (de Jesus Silva et al. 2019). The regression analyses support this finding, showing significant correlation coefficients ranging from 0.9161 to 0.9918. The *n*‐value for the CCS was notably lower at 0.0165. At the same time, the *n*‐values for the vegan formulations varied between 0.0192 and 0.0464, indicating more pronounced pseudoplastic behavior in the vegan chocolate spreads (Bascuas et al. 2021). The consistency coefficients (*K*‐values) of the various spreads highlight the relative viscosity across formulations, with *K*‐values ranging from 4.2437 to 5.8164 dyn/cm^2^ for the vegan samples, compared to a *K*‐value of 4.2437 dyn/cm^2^ for the CCS. This suggests greater viscosity in SVCS and LVCS, which may arise from enhanced water and oil binding capacities in these plant‐based ingredients (Gorin et al. 2022). Such binding dynamics likely contribute to a more complex network of interactions among the constituent particles, increasing the overall viscosity of the spreads.

Figure 4 reveals the flow behavior of a novel vegan chocolate spread sample. The maximum shear stress was recorded in the LVCS samples, reaching 373.81 Pa. In contrast, the CCS and the COCS recorded minimum shear stresses of 75.24 and 86.36 Pa, respectively. The RVCS and SVCS samples displayed shear stresses of 340.48 and 3.40 Pa, respectively. These findings suggest that adding oleogel does not significantly increase shear stress compared to the control, indicating that oleogel may provide a smoother texture without compromising spreadability (Pușcaș et al. 2022). Conversely, vegan chocolate spreads, especially those made with SVCS and LVCS, showed higher shear stress values. This increase can be attributed to the higher fiber and protein content in plant‐based milk powders, which enhance the thickening properties of the spread (Quispe‐Sánchez et al. 2022).

Regression analyses of the natural logarithm of shear stress against the natural logarithm of shear rate demonstrated a significant correlation for the investigated parameters of vegan chocolate spread samples, with a correlation coefficient (*R* ^2^) ranging from 0.9161 to 0.9918, as presented in Table S1. The *n*‐value for the control chocolate spread was lower (0.0165), while the *n*‐values for the tested vegan chocolate spreads ranged from 0.0192 to 0.0464. The low *n*‐values indicate a complete non‐Newtonian behavior of the tested samples. The results showed that RVCS and SVCS samples exhibited greater pseudo‐plasticity than the CCS, COCS, and LVCS samples.

The vegan chocolate spread samples showed consistency coefficients (*K*‐values) between 4.2437 and 5.8164 dyn/cm^2^, while the control chocolate spread sample had a lower *K*‐value of 4.2437 dyn/cm^2^. This suggests that the SVCS and LVCS samples had a more viscous consistency than the CCS sample. The soy milk powder and lentil milk powder bind more water and oil within the matrix of the vegan chocolate spreads, likely contributing to a more complex network of interactions among particles, resulting in increased viscosity (Lončarević et al. 2019). Several factors, including fat content, particle size, and the presence of emulsifiers, influence the rheological behavior of chocolate spreads. Cooney et al. noted that the viscosity of chocolate can vary significantly depending on the formulation, with higher fat content generally leading to lower viscosity (Cooney et al. 2024).

Oleogel in formulations may alter flow properties by creating a structured network that affects how the spread behaves under shear (Fayaz et al. 2017). Data indicate a trend of decreasing viscosity with increasing shear rate for all samples, which is characteristic of non‐Newtonian fluids. This behavior suggests that the chocolate spreads exhibit shear‐thinning properties, where viscosity decreases with increased shear, making them easier to spread (Poliński et al. 2021). The higher viscosity values observed in RVCS and LVCS formulations may present challenges in terms of spreadability, potentially affecting consumer preferences, particularly among those seeking a smooth, easily spreadable product (Lončarević et al. 2021).

### 3.9 Texture Profile Analysis

The texture profile analysis of novel vegan chocolate spreads reveals significant differences in textural attributes compared to a CCS. The parameters measured—firmness, springiness, cohesiveness, gumminess, spreadability, and adhesiveness—are critical for consumer acceptance and product quality in food science (Table 5). The control chocolate spread exhibited a firmness of 1.10 N. In contrast, the vegan chocolate spreads displayed varying degrees of firmness, with the RVCS being the least firm at 0.90 N. This indicates that the formulation of vegan spreads, particularly those using rice milk, may lead to a softer texture, influencing consumer preference depending on the product's intended use (Goktas et al. 2021; McGill and Hartel 2018). The springiness of the spreads varied, with the chocolate spread containing oleo‐gel (COCS) showing the highest springiness at 0.99 mm, suggesting a more elastic texture compared to the control spread. This elasticity is crucial as it can enhance the sensory experience of the product, making it more appealing to consumers (Azevedo et al. 2017). Cohesiveness, which reflects a product's ability to hold together, was consistent across the samples, all displaying a value of approximately 0.38, except for the lentil milk chocolate spread (LVCS), which had a higher cohesiveness of 0.69. This increased cohesiveness in LVCS may contribute to a more stable structure, potentially enhancing its appeal as a spreadable product (Beegum et al. 2022; Borges et al. 2024).

**TABLE 5:** Texture profile of novel vegan chocolate spreads and chocolate spread (control).

Sample	Firmness (*N*)	Springiness (mm)	Cohesiveness	Gumminess (*N*)	Spreadability (N*mm)	Adhesiveness (*N*)
CCS	1.10^b^ ± 0.10	0.56^c^ ± 0.02	0.38^b^ ± 0.01	0.41^c^ ± 0.01	0.23^d^ ± 0.02	−0.30^bc^ ± 0.05
COCS	1.00^b^ ± 0.20	0.59^b^ ± 0.01	0.38^b^ ± 0.02	0.38^d^ ± 0.01	0.37^c^ ± 0.05	−0.25^b^ ± 0.02
RVCS	0.90^b^ ± 0.10	0.57^bc^ ± 0.02	0.38^b^ ± 0.01	0.34^e^ ± 0.02	0.20^e^ ± 0.04	−0.20^a^ ± 0.01
SVCS	2.50^a^ ± 0.30	0.59^bc^ ± 0.03	0.32^c^ ± 0.03	0.80^a^ ± 0.03	0.47^b^ ± 0.01	−0.80^d^ ± 0.02
LVCS	1.00^b^ ± 0.20	0.99^a^ ± 0.08	0.69^a^ ± 0.04	0.69^b^ ± 0.05	0.68^a^ ± 0.09	−0.40^c^ ± 0.01

*Note:* Mean ± SD was used to display the data (*n* = 3). A substantial variation (*p* < 0.05) was indicated by distinct letters for each column.

Abbreviations: CCS, control chocolate spread; COCS, chocolate spread with oleo‐gel prepared as a butter alternative; RCLS, vegan chocolate spread made with brown lentil milk powder and oleo‐gel as alternatives to whole milk powder and butter; RVCS, vegan chocolate spread made with rice milk powder and oleo‐gel as alternatives to whole milk powder and butter; SVCS, vegan chocolate spread made with soy milk powder and oleo‐gel as alternatives to whole milk powder and butter.

Gumminess, a measure of the energy required to disintegrate semi‐solid food, was highest in the SVCS at 0.80 N, indicating that this formulation may provide a more substantial mouthfeel than others. This characteristic is crucial for consumer satisfaction, as it contributes to the overall eating experience (Yılmaz and Öz 2024). Spreadability, a vital attribute for chocolate spreads, was highest in the LVCS at 0.68 N*mm, suggesting that this formulation may be particularly desirable for consumers looking for easy‐to‐spread products. Conversely, the RVCS had the lowest spreadability at 0.20 N*mm, which could limit its application in specific culinary contexts (Popov‐Raljić et al. 2010). Adhesiveness, which indicates how well a product sticks to surfaces, was notably negative across all samples, with the SVCS showing the highest adhesiveness at −0.80 N. This negative value suggests that these spreads may not excessively adhere to surfaces, which could benefit ease of use (Taşoyan et al. 2023; Singh et al. 2022). The texture profile analysis indicates that the novel vegan chocolate spreads exhibit textural properties that differ significantly from traditional chocolate spreads. These differences are critical for product development and consumer acceptance as they influence the sensory experience associated with the product. The findings highlight the potential for formulating vegan spreads that meet consumer expectations for texture while providing alternative ingredients to traditional dairy‐based products (Lončarević et al. 2021).

### 3.10 Index of Oxidative Stability

**FIGURE 5:**
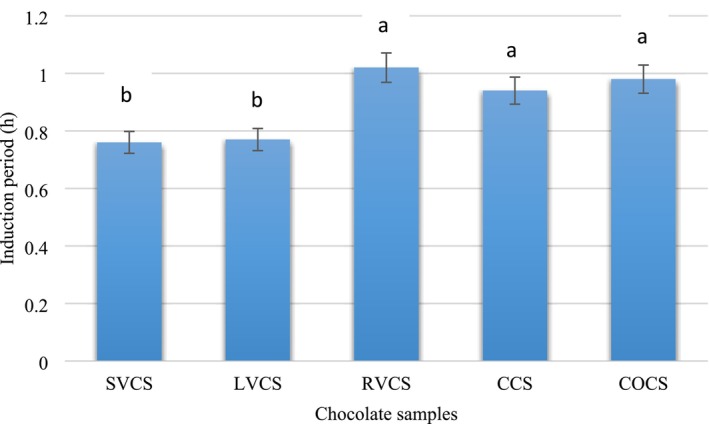
Shows how long Rancimat incubated the spreadable chocolate samples. CCS, Control chocolate spread; COCS, Chocolate spread with oleo‐gel prepared as a butter alternative; RCLS, Vegan chocolate spread made with brown lentil milk powder and oleo‐gel as alternatives to whole milk powder and butter; RVCS, Vegan chocolate spread made with rice milk powder and oleo‐gel as alternatives to whole milk powder and butter; SVCS, Vegan chocolate spread made with soy milk powder and oleo‐gel as alternatives to whole milk powder and butter; Mean ± SD was used to display the data (*n* = 3). A substantial variation (*p* < 0.05) was indicated by distinct letters for each column.

There are notable variations among the examined samples when it comes to the oxidative stability of the spreadable chocolate formulations, which is evaluated using the Rancimat technique throughout the induction period (IP). In comparison to the other formulations, RVCS formulation has the highest IP value of 1.02 h at 110°C, demonstrating improved oxidative stability (Figure 5). The spreadable chocolates prepared with LVCS and SVCS, on the other hand, had lower IP values of 0.77 and 0.76 h, respectively, which may indicate a greater vulnerability to oxidative degradation. While still less than SVCS, the IP of the traditional control formula (CCS) is 0.94 h, which is rather consistent.

These results emphasize how crucial ingredient choice is for extending shelf life; in particular, rice milk powder seems to have advantageous qualities that increase stability without sacrificing quality (Amagliani et al. 2016). Overall, the findings highlight the possibility of creating healthier vegan chocolate products with longer shelf lives by using different plant‐based proteins, which would increase customer acceptability and pleasure. This is consistent with other research that highlights the importance of oxidative stability in cocoa fats and how formulation variations may affect stability over time. According to studies, oleo gels are a viable substitute for conventional fats like cocoa butter since they may successfully avoid fat bloom and increase stability in the manufacturing of chocolate (Valdivia‐Culqui et al. 2024). The additional study supports the potential advantages of structured oils in food applications by showing that different oleo gelators may improve structural qualities and antioxidant capacity (Wang et al. 2023).

### 3.11 Sensory Acceptability

The sensory acceptability of novel vegan chocolate spreads compared to a CCS is a crucial aspect of product development, as it directly influences consumer preferences and market success (Table S2). The parameters evaluated in the sensory analysis include color, smoothness, taste, texture, aroma, appearance, and overall acceptability, which are vital for understanding how consumers perceive these products. The control chocolate spread exhibited high sensory scores across various attributes, with a firmness of 1.10 N and a taste rating of 9.17 ± 0.22, indicating a strong consumer preference for its texture and flavor profile. In contrast, the vegan chocolate spreads displayed lower scores, particularly the RVCS, which had a firmness of 0.90 N and lower ratings in smoothness (7.06 ± 0.21) and overall acceptability (7.50 ± 0.24). This suggests that while vegan formulations may offer health benefits, they may not yet meet the sensory expectations set by traditional chocolate spreads (Goktas et al. 2021). Among the vegan spreads, the COCS showed promising results, particularly in springiness and overall acceptability, comparable to the control spread. This indicates that incorporating oleo‐gel may enhance the sensory attributes of vegan chocolate spreads, making them more appealing to consumers (Nigussie et al. 2021). The LVCS also demonstrated higher cohesiveness and spreadability, which are critical for achieving a desirable texture and mouthfeel (Lee et al. 2024).

The sensory evaluation results indicate that while the control spread is preferred for its taste and texture, vegan alternatives, especially those formulated with oleo‐gel and lentil milk, show potential for improvement in sensory attributes. The findings align with previous research, emphasizing the importance of texture and mouthfeel in consumer acceptance of chocolate products. For instance, studies have shown that adding certain ingredients can significantly enhance the sensory profile of chocolate, making it more appealing to consumers (Pușcaș et al. 2022). The sensory acceptability analysis of novel vegan chocolate spreads reveals that while they differ significantly from traditional chocolate spreads in various attributes, there is potential for improvement through careful formulation. Incorporating ingredients like oleo‐gel and lentil milk can enhance the sensory experience, making these products more competitive. Ongoing research and development are essential to optimize these formulations to meet consumer expectations for taste, texture, and overall quality (Ng et al. 2020).

## 4 Conclusion

This study successfully demonstrated the feasibility of developing a nutritionally enhanced, plant‐based chocolate spread using plant‐based milk powders, nano‐liposomal Vitamin D3, and an oleogel to balance omega fatty acids. The resulting spreads offer a vegan alternative to traditional chocolate spreads without sacrificing sensory appeal. Encapsulation of Vitamin D3 in nanoliposomes proved to be an effective strategy for enhancing its potential bioavailability and stability, while the incorporation of the carefully formulated oleogel significantly improved the overall fatty acid profile. These findings suggest that plant‐based chocolate spreads can be designed to address specific nutritional deficiencies and cater to the growing demand for healthier, more sustainable food options. Further research is recommended to investigate the sensory properties of the optimized spreads, consumer acceptance, and long‐term stability, as well as in vivo studies, which will provide evidence for improved bioavailability. This work provides a foundation for developing functional plant‐based confectionery products with improved nutritional profiles.

## Author Contributions

**Dina E. H. Azab:** conceptualization (equal), data curation (equal), investigation (equal), writing – original draft (equal), writing‐review and editing (equal). **Ebtehal A. Altamim:** conceptualization (equal), data curation (equal), writing – original draft (equal), writing – review and editing (equal). **Tarek N. Soliman:** conceptualization (equal), data curation (equal), methodology (equal), writing – original draft (equal). **Sahar A. Nasser:** conceptualization (equal), formal analysis (equal), writing – original draft (equal). **Hamdy A. Zahran:** conceptualization (equal), data curation (equal), supervision (equal), writing – original draft (equal), writing – review and editing (equal).

## Ethics Statement

The authors have nothing to report.

## Conflicts of Interest

The authors declare no conflicts of interest.

## Supporting information


**Tables S1–S2:** fsn370827‐sup‐0001‐TablesS1‐S2.docx.

## Data Availability

No data that has been used is confidential.
