# Supplementary material for: Development of a Plant‐Based Chocolate Spread With Enhanced Vitamin D3 Bioavailability and Balanced Omega Fatty Acids
Source: Food Sci Nutr. 2025 Sep 7;13(9):e70827. doi: 10.1002/fsn3.70827 (PMC12415268; doi:10.1002/fsn3.70827)
Supplement: Supplementary file 1 — Tables S1–S2: fsn370827‐sup‐0001‐TablesS1‐S2.docx. [file FSN3-13-e70827-s001.docx]

**Supplementary data**

**SI:** Rheological parameters of novel vegan chocolate spread samples

| Samples | Flow behavior N-Index | Consistency coefficient (K) Dynes.S^n^/cm^2^ | R^2^ |
| --- | --- | --- | --- |
| CCS | 0.0165 | 4.2437 | 0.9918 |
| COCS | 0.0384 | 4.2761 | 0.9698 |
| RVCS | 0.0464 | 4.6934 | 0.9523 |
| SVCS | 0.0325 | 5.6472 | 0.9222 |
| LVCS | 0.0192 | 5.8164 | 0.9161 |

*CCS: control chocolate spread; COCS: chocolate spread with oleo-gel prepared as a butter alternative; RVCS: vegan chocolate spread made with rice milk powder and oleo-gel as alternatives to whole milk powder and butter; SVCS: vegan chocolate spread made with soy milk powder and oleo-gel as alternatives to whole milk powder and butter; LVCS: vegan chocolate spread made with brown lentil milk powder and oleo-gel as alternatives to whole milk powder and butter.*

**S2.** Sensory acceptability of novel vegan chocolate spreads and chocolate spreads (control)

| Samples | Color | Smoothness | Taste | Texture | Aroma | Appearance | Overall acceptability |
| --- | --- | --- | --- | --- | --- | --- | --- |
| CCS | 8.89±0.20^b^ | 8.94± 0.15^b^ | 9.17±0.22^a^ | 9.11±0.16^a^ | 8.84±0.08^a^ | 8.67± 0.14^b^ | 8.67± 0.12^a^ |
| COCS | 8.89±0.20^b^ | 8.94± 0.15^b^ | 9.02±0.22^a^ | 9.00±0.16^a^ | 8.32±0.08^a^ | 8.55± 0.14^b^ | 8.17± 0.12^a^ |
| RVCS | 9.11±0.16^a^ | 9.00±0.12^a^ | 9.39±0.14^a^ | 9.00±0.20^a^ | 8.92±0.06^a^ | 9.06± 0.10^a^ | 9.11± 0.14^a^ |
| SVCS | 8.10±0.15^b^ | 8.15±0.13^b^ | 8.60±0.12^b^ | 8.50±0.13^b^ | 8.38±0.23^a^ | 8.45±0.21^b^ | 7.85± 0.18^b^ |
| LVCS | 7.94±0.49^b^ | 7.06±0.21^c^ | 7.78±0.19^b^ | 8.17±0.25^b^ | 7.83±0.12^b^ | 8.22± 0.12^b^ | 7.50±0.24^b^ |

*The results were expressed as mean ± SE (n-values = 3), with different letters in the same column indicating the significant difference between samples.* *CCS: control chocolate spread; COCS: chocolate spread with oleo-gel prepared as a butter alternative; RVCS: vegan chocolate spread made with rice milk powder and oleo-gel as alternatives to whole milk powder and butter; SVCS: vegan chocolate spread made with soy milk powder and oleo-gel as alternatives to whole milk powder and butter; RCLS: vegan chocolate spread made with brown lentil milk powder and oleo-gel as alternatives to whole milk powder and butter.*
